# Putrescine-functionalized carbon quantum dot (put-CQD) nanoparticles effectively prime grapevine (*Vitis vinifera* cv. ‘Sultana’) against salt stress

**DOI:** 10.1186/s12870-021-02901-1

**Published:** 2021-02-27

**Authors:** Gholamreza Gohari, Sima Panahirad, Mostafa Sadeghi, Ali Akbari, Elnaz Zareei, Seyed Morteza Zahedi, Mohammad Kazem Bahrami, Vasileios Fotopoulos

**Affiliations:** 1 Department of Horticultural Sciences, Faculty of Agriculture, University of Maragheh, Maragheh, Iran; 2 Department of Horticultural Sciences, Faculty of Agriculture, University of Tabriz, Tabriz, Iran; 3 Solid Tumor Research Center, Cellular and Molecular Medicine Institute, Urmia University of Medical Sciences, Urmia, Iran; 4 Department of Biology, Faculty of Sciences, University of Maragheh, Maragheh, Iran; 5 Department of Agricultural Sciences, Biotechnology and Food Science, Cyprus University of Technology, Limassol, Cyprus

**Keywords:** Carbon quantum dots, Grapevine, Nanotechnology, Priming, Abiotic stress

## Abstract

**Background:**

Salinity is an important global problem with destructive impacts on plants leading to different biochemical and metabolic changes in plants through induced oxidative stress that disturbs metabolism, growth, performance and productivity of plants. Given that putrescine (Put) and carbon quantum dots (CQDs), individually, have promising effects in different plant processes, the idea of their combination in a nano-structure “Put-CQD” lead to its synthesis to evaluate the potential exertion of synergistic effects. The current study aimed to investigate the application of newly-synthesized nanoparticles (NPs) consisting of CQDs and Put in grapevine (*Vitis vinifera* cv. ‘Sultana’) under salinity stress conditions. For this purpose, Put, CQDs and Put-CQD NPs at 5 and 10 mg L^− 1^ concentrations were applied as chemical priming agents in ‘Sultana’ grapevine 48 h prior salinity stress imposition (0 and 100 mM NaCl).

**Results:**

Salinity significantly decreased (*P* ≤ 0.05) morphological parameters, photosynthetic pigments, chlorophyll fluorescence parameters and membrane stability index. In addition, salinity enhanced MDA, H_2_O_2_, proline content and antioxidant enzyme activity. Results revealed that Put-CQD NPs, particularly at 10 mg L^− 1^ concentration, alleviated the destructive impacts of salinity stress by improving leaf fresh and dry weights, K^+^ content, photosynthetic pigments, chlorophyll fluorescence and SPAD parameters, proline content, total phenolics and antioxidant enzymatic activities (CAT, APX, GP and SOD), while decreasing Na^+^ content, EL, MDA and H_2_O_2_ levels.

**Conclusion:**

To conclude, Put-CQD NPs represent an innovative priming treatment that could be effectively applied on grapevine to improve plant performance under salinity stress conditions.

## Background

Abiotic stress factors such as salinity and drought are affecting most agricultural lands, limiting plant distribution in habitats. Salinity stress, a major environmental restrain and a key global climate change-related problem, causes negative impacts on performance and yield of plants [1, 2]. Substantial disorders in morphological, physiological, nutritional and biochemical characteristics, ion toxicity or imbalance (Na^+^ and Cl^−^) and osmotic stress are some of the main salinity effects in plants [3]. As a result, salinity stress leads to numerous biochemical and metabolic changes resulting in oxidative stress in plants [2, 4].

Chemical priming is amongst several approaches employed to deal with stresses like salinity, as an advantageous technique to increase plant tolerance to different stresses [5, 6], before their occurrence [7]. Natural compounds such as amino acids (e.g. proline and glycine-betaine [8];) and hormones (e.g. salicylic acid [9];), as well as synthetic molecules (e.g. NOSH-aspirin [10];), are amongst a number of materials with the potential of acting as priming agents against stresses. Moreover, certain chemicals (e.g., [11]) and nanoparticles (NPs) (e.g., [12, 13]) could ameliorate the negative effects of salinity stress through priming.

Abundant benefits such as superior performance of chemicals via improved nano-structure are proposed by nanotechnology, which then cause reduction in their environmental load via chemical priming application [14]. Therefore, their application represents a promising strategy for agricultural industries [15]. NPs have great impact on plant growth and development, as well as on plant tolerance to abiotic stresses through their effectiveness in ROS detoxification [16]. Furthermore, photosynthesis, as a cellular process sensitive to abiotic stresses, could be protected by NP application by diminishing osmotic and oxidative stresses [14]. Subsequently, NP application has been attracting increasing attention in this regard. Quantum dot (QD) NPs are 2–10 nm in size, giving them the role of a carrier and size-dependent essential properties. They are zero dimensional and semiconductor NPs with quantization of energy [17]. In addition, QDs are extensively applied in biological studies for subcellular labeling and imaging through their distinctive fluorescent characteristics [18]. Carbon QDs (CQDs), as a new generation of QD NPs in which carbonic compounds contain oxygen, show somewhat different properties than carbon-based NPs [19]. CQD NPs are less than 10 nm in size (like other QDs) with round shape giving them unique physicochemical properties (e.g., low or no toxicity and high water solubility, biocompatibility and biodegradability) with enormous range of usage [20]. Some studies have demonstrated advantageous impacts of different QDs in plants; graphene QDs caused an increase in growth parameters (e.g., leaves, roots, shoots, flowers and fruits) of treated garlic and coriander seeds [21]. CQD NPs enhanced rice yield due to increased RuBisCO enzyme activity. In addition, they improved seed germination, root elongation, carbohydrate production and resistance to diseases all through enhanced thionin gene expression [22].

Putrescine (Put), as one of major polyamines (PAs), has crucial functions in plant growth and differentiation and also response to stresses with salt stress in particular [1, 23–25]. Put stabilizes biological membranes and macromolecular structures of cells [26]. With low molecular weight and polycationic nature [27, 28], Put plays important roles in numerous physiological and developmental processes such as cell division, rhizogenesis, embryogenesis, senescence, floral development and fruit setting and ripening. It is worth stating that Put concentration increases under stress conditions to enhance plant tolerance to the stressor [26]. Such an increasing trend in Put content was reported in plants under salt stress [29]. Put application enhances stress tolerance by stabilizing membrane and cellular structures, scavenging free radicals, modulating ion channels, maintaining the cation-anion balance and energizing cells via stimulating of ATP synthesis [24, 28]. Therefore, exogenous Put treatment could be considered as a typical attempt to improve plant performance under salinity, mostly by enhancing photosynthetic efficiency and preventing chlorophyll loss [1]. Plant species, duration and intensity of stress, developmental stage of plant tissues and applied treatments could affect Put content of plants under stress conditions. Response to the stress condition by Put demonstrates its role as a signaling molecule [26]. Put could reverse growth inhibition caused by stress, decrease cell membrane damage, lipid peroxidation, ROS accumulation, increase in Na^+^ and Cl^−^ and loss of chlorophyll, and also increase expression of osmotically responsive genes, antioxidant enzymatic activities, non-enzymatic compounds and compatible osmolytes [29]. Shu et al. [1] noticed that Put treatment increased Put content in plants under salinity conditions. Put application decreases stress and lipid peroxidation damage of plant under salt stress by improving plant growth and antioxidant enzyme activity, inhibiting Na^+^ and Cl^−^ uptake and accelerating accumulation of K^+^, Ca^2+^ and Mg^2+^ [30].

Grapevine (*Vitis vinifera* L.) is considered as one of the most important, added-value agricultural products cultivated worldwide, consequently with significant economic importance [31]. Grapevine, relatively sensitive to salt stress, suffers significant losses in growth and productivity and also fruit quality under salinity conditions [32].

Considering the protective properties of CQD NPs and Put when individually applied, especially at lessening destructive effects of salinity stress, conjugating Put with CQD NPs (Put-CQD NPs) could potentially improve entrance of Put inside plant cells and thus improve its efficiency, particularly at lower doses. Consequently, after synthesizing Put-CQD NPs, NPs were applied as priming agents in grapevine (*Vitis vinifera* cv. ‘Sultana’) to alleviate the undesirable impacts of salt stress conditions, representing the first report of its kind to our knowledge.

## Results

### Put-CQDs synthesis and characterization

**Fig. 1:**
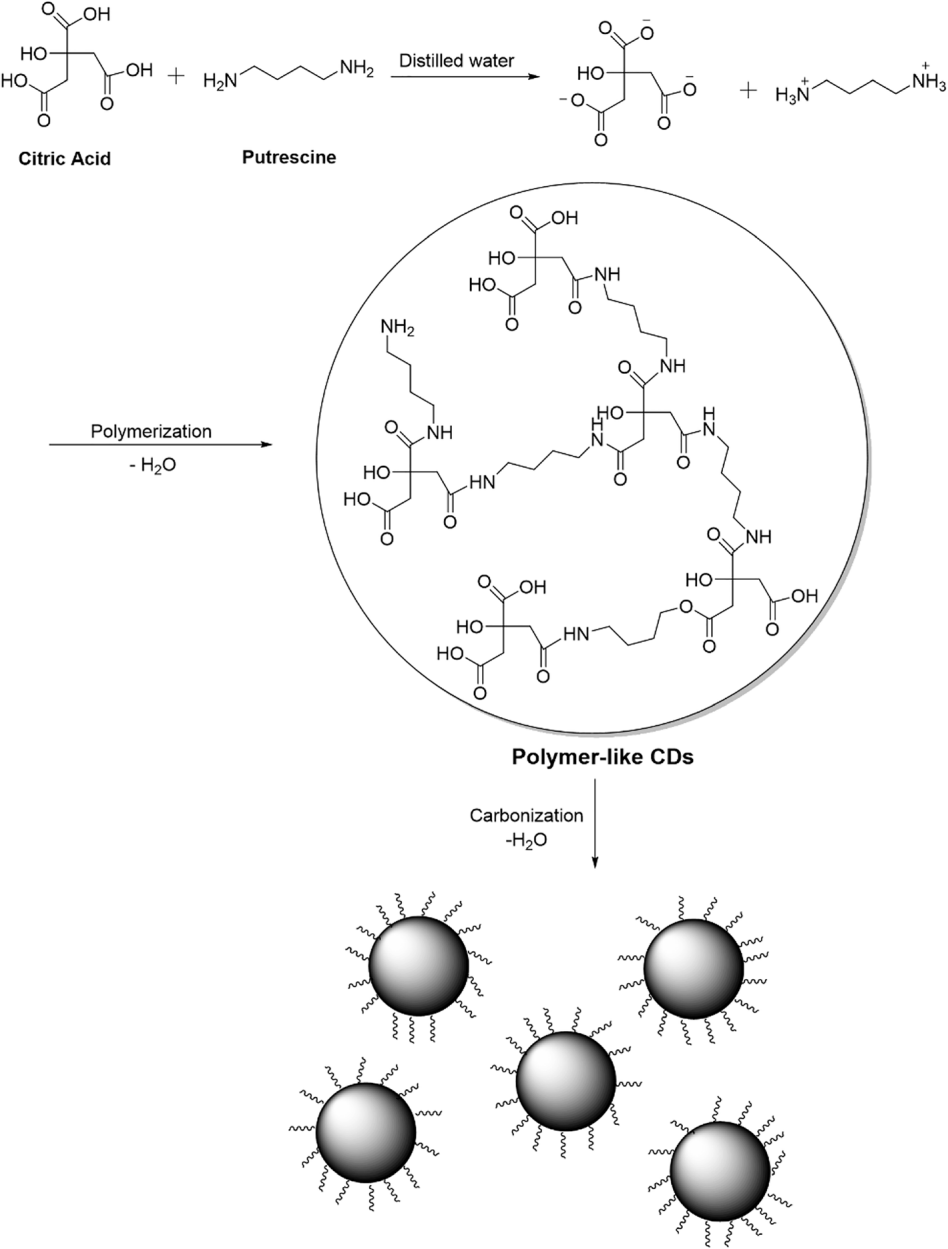
A synthetic step of Put-CQD NPs: from ionization to condensation, polymerization, and carbonization

**Fig. 2:**
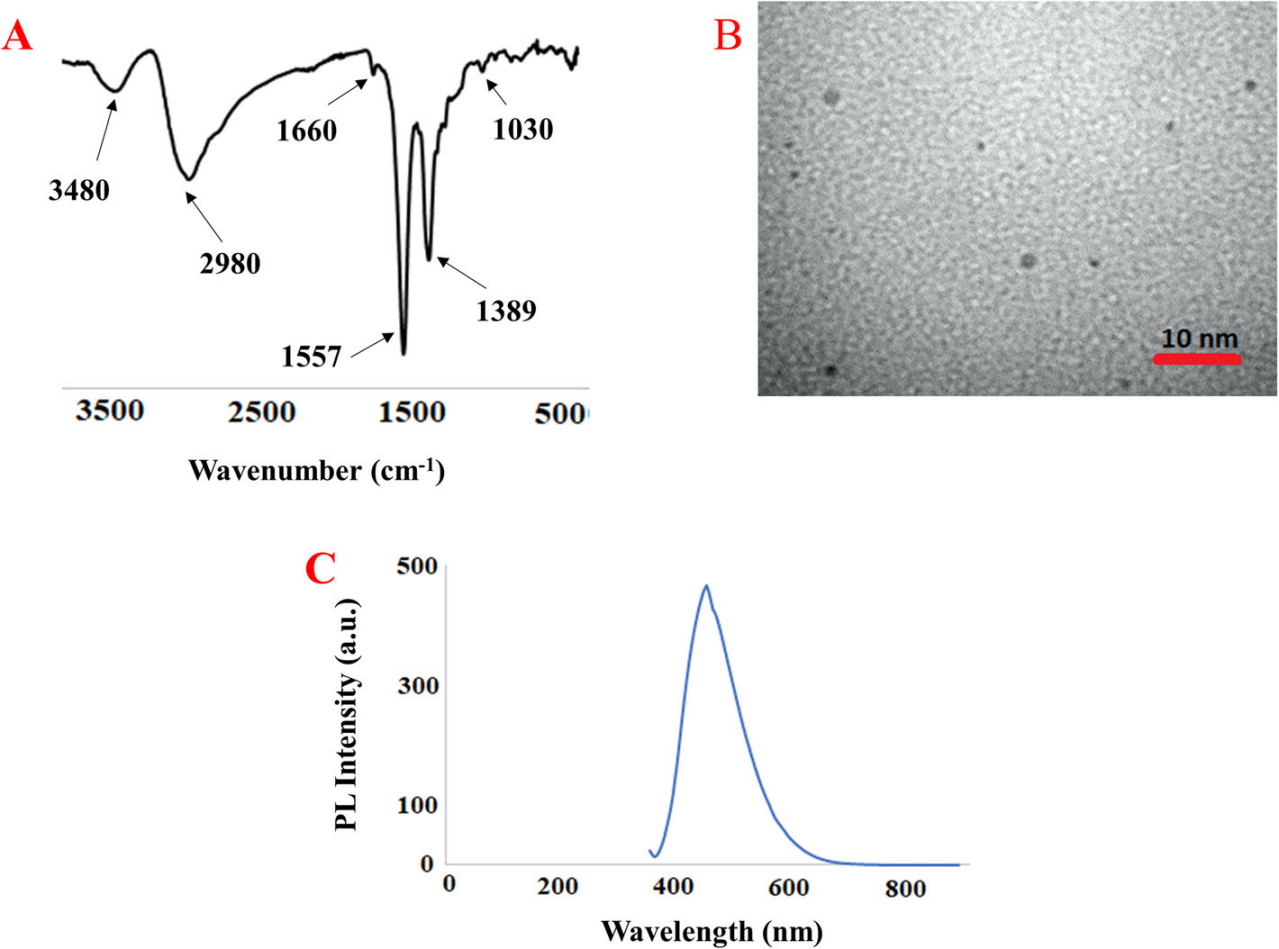
FTIR spectrum (**a**), TEM image (**b**) and PL spectrum (**c**) of synthesized Put-CQD NPs

One-pot and easy hydrothermal method was used in the preparation of Put-CQD NPs. Citric acid is the most commonly used carbon source which could be utilized alone or with other functionalized amines for CQDs preparation. As can be seen from Fig. 1, the carboxylic acid groups of citric acid firstly condensed with the amine groups of putrescines leading to form polymer-like CQDs, which were then carbonized to form the CDs. The structure composition and morphology of Synthesized Put-CQD NPs were characterized using FTIR and TEM analysis. In the FTIR spectrum of Put-CQD NPs (Fig. 2a), characteristic bands at 3480 cm^− 1^ and 2980 cm^− 1^ corresponded to the stretching vibrations of O–H and C–H, respectively. The sharp bands at 1557 cm^− 1^ and 1389 cm^− 1^ could be related to bending vibration of N–H and C–NH–C, respectively. Moreover, C=O and C–N stretching vibration could be seen at 1660 cm^− 1^ and 1030 cm^− 1^. Figure 2b illustrates TEM image of Put-CQD NPs. As it could be seen, the synthesized Put-CQD NPs have uniform dispersion without significant aggregation and particle size in the range of 3–5 nm. Furthermore, the PL spectrum shows blue fluorescence with λ_max_ at 440 nm under 360 nm UV-light (Fig. 2c).

### Leaf FW and DW

Salinity negatively affected leaf FW and DW. Leaf FW increased significantly (*P* ≤ 0.05) by 10 mg L^− 1^ Put and 5 and 10 mg L^− 1^ Put-CQD NPs; however, the other treatments had no effect or decreased the weight under control conditions. All treatments enhanced leaf DW under control conditions. Under salt stress condition, 10 mg L^− 1^ CQD NPs and 5 and 10 mg L^− 1^ Put-CQD NPs increased leaf FW, while the other priming treatments exerted no effect compared with unprimed grapevines under salinity. All priming treatments enhanced leaf DW under salinity condition. Under both conditions, 10 mg L^− 1^ Put-CQD NPs could be considered as the optimal priming treatment (Table 1).

**Table 1:** Effect of different concentrations of Put, CQDs, and Put-CQD NPs on leaf fresh (FW) and dry (DW) weights, Na^+^ and K^+^ concentrations and ratio of *Vitis vinifera* cv. ‘Sultana’ under salinity stress (CQDs, carbon quantum dots; Put, putrescine, Put-CQD NPs, carbon quantum dots functionalized by putrescine nanoparticles). Different letters indicate significant differences based on Duncan’s post-hoc analysis at *P* ≤ 0.05

Treatments	Leaf FW (g)	Leaf DW (g)	Na^**+**^ (mM kg^**− 1**^)	K^**+**^ (mM kg^**− 1**^)	Na^**+**^/K^**+**^
NaCl (0 mM) + No Treatment	83.61 ± 0.21^de^	7.15 ± 0.03^e^	0.212 ± 0.019^ef^	5.64 ± 0.91^bc^	0.037 ± 0.003^f^
NaCl (0 mM) + Put 5 mg L^− 1^	85.23 ± 0.08^d^	8.51 ± 0.01^d^	0.185 ± 0.014^g^	5.72 ± 0.61^bc^	0.032 ± 0.009^fg^
NaCl (0 mM) + Put 10 mg L^− 1^	87.54 ± 0.15^c^	8.92 ± 0.08^cd^	0.173 ± 0.013^gh^	5.87 ± 0.83^b^	0.029 ± 0.005^g^
NaCl (0 mM) + CQDs 5 mg L^− 1^	81.39 ± 0.41^f^	9.55 ± 0.07^c^	0.202 ± 0.017^f^	4.96 ± 0.43^c^	0.048 ± 0.007^e^
NaCl (0 mM) + CQDs 10 mg L^− 1^	85.08 ± 0.38d^d^	10.06 ± 0.09^bc^	0.191 ± 0.011^fg^	5.51 ± 0.67^bc^	0.034 ± 0.004^fg^
NaCl (0 mM) + Put-CQD NPs 5 mg L^− 1^	89.87 ± 0.29^b^	10.15 ± 0.05^b^	0.171 ± 0.008^gh^	6.16 ± 0.89^ab^	0.027 ± 0.009^g^
NaCl (0 mM) + Put-CQD NPs 10 mg L^− 1^	92.09 ± 0.61^a^	11.01 ± 0.03^a^	0.163 ± 0.016^h^	7.08 ± 0.69^a^	0.023 ± 0.004^h^
NaCl (100 mM) + No Treatment	65.36 ± 0.2^ij^	4.01 ± 0.04^i^	0.461 ± 0.016^a^	2.58 ± 0.28^f^	0.178 ± 0.006^a^
NaCl (100 mM) + Put 5 mg L^− 1^	65.05 ± 0.19^j^	4.85 ± 0.03^h^	0.355 ± 0.019^b^	3.12 ± 0.81^e^	0.113 ± 0.008^b^
NaCl (100 mM) + Put 10 mg L^− 1^	68. 23 ± 0.08^ij^	5.17 ± 0.09^g^	0.258 ± 0.006^de^	3.89 ± 0.97^de^	0.066 ± 0.005^d^
NaCl (100 mM) + CQDs 5 mg L^− 1^	71.02 ± 0.19^i^	5.82 ± 0.02^fg^	0.321 ± 0.017^bc^	2.74 ± 0.61^ef^	0.117 ± 0.003^b^
NaCl (100 mM) + CQDs 10 mg L^− 1^	74.91 ± 0.27^h^	6.07 ± 0.06^f^	0.309 ± 0.014^c^	2.98 ± 0.81^ef^	0.103 ± 0.007^c^
NaCl (100 mM) + Put-CQD NPs 5 mg L^− 1^	79.15 ± 0.43^g^	7.88 ± 0.06^de^	0.284 ± 0.007^d^	4.13 ± 0.65^d^	0.068 ± 0.008^d^
NaCl (100 mM) + Put-CQD NPs 10 mg L^− 1^	82.34 ± 0.31^e^	7.97 ± 0.04^de^	0.229 ± 0.009^e^	3.93 ± 0.75^de^	0.058 ± 0.004^de^

### Ionic homeostasis

Salinity significantly decreased (*P* ≤ 0.05) K^*+*^ content and increased Na^*+*^ content and Na^*+*^/K^*+*^ ratio. Under non saline conditions, all treatments lowered Na^*+*^ content compared with the control and K^*+*^ content was only enhanced in grapevine primed by 10 mg L^− 1^ Put-CQD NPs. Consequently, all priming treatments increased the Na^+^/K^+^ ratio, with the exception of 5 mg L^− 1^ put and 10 mg L^− 1^ CQDs which had no effect compared with the control. Under salinity conditions, all treatments decreased Na^*+*^ content and Na^+^/K^+^ ratios, while Put and Put-CQD NPs treatments (5 and 10 mg L^− 1^) increased K^+^ content. Put-CQD NPs at 10 mg L^− 1^ concentration demonstrated the best results for K^*+*^, Na^*+*^ and Na^*+*^/K^*+*^ ratio under both conditions (Table 1).

### Physiological parameters

Chl *a*, *b* and carotenoid content were decreased by salinity. Significant increase (*P* ≤ 0.05) in Chl *a* and *b* content was observed following Put and Put-CQD NPs priming treatments; all priming treatments enhanced carotenoid content under control conditions compared with the control. Under stress conditions, all treatments significantly increased Chl *a, b* and carotenoid content. Put-CQD NPs at 10 mg L^− 1^ lead to the highest value for Chl *a, b* and carotenoid levels under both control and stress conditions. SPAD value was negatively affected by salinity. With the exception of CQDs at 10 mg L^− 1^ which showed no significant difference to the control, other treatments enhanced SPAD values under non-stress conditions. Increase in SPAD values was additionally achieved by Put (5 and 10 mg L^− 1^), CQDs (10 mg L^− 1^) and Put-CQD NPs (5 and 10 mg L^− 1^) priming treatments under salt stress conditions. The highest SPAD value was recorded at 10 mg L^− 1^ Put-CQD NPs-primed grapevines (Table 2).

**Table 2:** Effect of different concentrations of Put, CQDs and Put-CQD NPs on photosynthetic pigments and SPAD index of *Vitis vinifera* cv. ‘Sultana’ under salinity stress (CQDs, carbon quantum dots; Put, putrescine, Put-CQD NPs, carbon quantum dots functionalized by putrescine nanoparticles). Different letters indicate significant differences based on Duncan’s post-hoc analysis at P ≤ 0.05

Treatments	Chl ***a*** (mg g^**− 1**^ FW)	Chl ***b*** (mg g^**− 1**^ FW)	Carotenoids (mg g^**− 1**^ FW)	SPAD
NaCl (0 mM) + No Treatment	2.039 ± 0.05^ef^	0.579 ± 0.002^cd^	0.521 ± 0.003^e^	26.86 ± 0.73^c^
NaCl (0 mM) + Put 5 mg L^− 1^	2.228 ± 0.02^c^	0.657 ± 0.008^b^	0.633 ± 0.012^bc^	29.38 ± 0.83^ab^
NaCl (0 mM) + Put 10 mg L^− 1^	2.317 ± 0.12^bc^	0.682 ± 0.011^ab^	0.662 ± 0.009^b^	29.91 ± 0.54^ab^
NaCl (0 mM) + CQDs 5 mg L^− 1^	1.797 ± 0.09^fg^	0.538 ± 0.009^d^	0.584 ± 0.011^cd^	28.13 ± 0.91^b^
NaCl (0 mM) + CQDs 10 mg L^− 1^	1.983 ± 0.01^ef^	0.582 ± 0.023^cd^	0.604 ± 0.013^c^	25.83 ± 0.95^cd^
NaCl (0 mM) + Put-CQD NPs 5 mg L^− 1^	2.324 ± 0.08^b^	0.697 ± 0.016^b^	0.649 ± 0.008^bc^	29.26 ± 0.38^ab^
NaCl (0 mM) + Put-CQD NPs 10 mg L^− 1^	2.426 ± 0.05^a^	0.723 ± 0.008^a^	0.721 ± 0.009^a^	31.82 ± 0.77^a^
NaCl (100 mM) + No Treatment	1.482 ± 0.12^h^	0.357 ± 0.014^f^	0.358 ± 0.007^f^	19.63 ± 0.14^g^
NaCl (100 mM) + Put 5 mg L^− 1^	1.718 ± 0.07^fg^	0.477 ± 0.021^e^	0.597 ± 0.012^cd^	21.7 ± 0.54^ef^
NaCl (100 mM) + Put 10 mg L^− 1^	1.891 ± 0.11^f^	0.534 ± 0.016^d^	0.612 ± 0.018^c^	22.7 ± 1.12^e^
NaCl (100 mM) + CQDs 5 mg L^− 1^	1.686 ± 0.09^g^	0.456 ± 0.009^e^	0.523 ± 0.014^de^	20.13 ± 0.82^fg^
NaCl (100 mM) + CQDs 10 mg L^− 1^	1.751 ± 0.08^fg^	0.461 ± 0.015^e^	0.542 ± 0.009^d^	21.4 ± 0.61^f^
NaCl (100 mM) + Put-CQD NPs 5 mg L^− 1^	2.052 ± 0.01^e^	0.596 ± 0.022^c^	0.648 ± 0.019^bc^	22.23 ± 1.24^ef^
NaCl (100 mM) + Put-CQD NPs 10 mg L^− 1^	2.141 ± 0.08^d^	0.586 ± 0.008^cd^	0.663 ± 0.008^b^	24.2 ± 0.43^d^

Chlorophyll fluorescence parameters were significantly negatively affected after imposing salinity. ^Fv^/_Fm_ was increased by all treatments, with the sole exception of 5 mg L^− 1^ CQDs priming treatment which showed no difference to unprimed, unstressed samples. All priming treatments positively affected ^Fv^/_Fm_ under stress conditions. Put and Put-CQD NPs priming treatments increased ^Fv^/_Fo_ parameter under control and stress conditions. Regarding Y (II) parameter, Put-CQD NP treatments (5 and 10 mg L^− 1^) leads to its significant increase (*P* ≤ 0.05) under control conditions. Under salinity stress, all treatments enhanced Y (II) parameter. Considering all parameters, Put-CQD NPs at 10 mg L^− 1^ concentration represented the optimal treatment under both control-stress and stress conditions (Table 3).

**Table 3:** Effect of different concentrations of Put, CQDs, and Put-CQD NPs on chlorophyll fluorescence parameters of *Vitis vinifera* cv. ‘Sultana’ under salinity stress (CQDs, carbon quantum dots; Put, putrescine, Put-CQD NPs, carbon quantum dots functionalized by putrescine nanoparticles). Different letters indicate significant differences based on Duncan’s post-hoc analysis at *P* ≤ 0.05

Treatments	Fv	Fm	Fo	^**Fv**^/_**Fm**_	^**Fv**^/_**Fo**_	Y (II)
NaCl (0 mM) + No Treatment	2.455 ± 0.09^c^	2.631 ± 0.08^d^	1.072 ± 0.08^ab^	0.933 ± 0.03^d^	2.291 ± 0.01^d^	0.674 ± 0.01^c^
NaCl (0 mM) + Put 5 mg L^− 1^	2.567 ± 0.03^ab^	2.252 ± 0.07^e^	1.069 ± 0.04^ab^	1.139 ± 0.01^c^	2.401 ± 0.04^c^	0.672 ± 0.02^c^
NaCl (0 mM) + Put 10 mg L^− 1^	2.579 ± 0.06^ab^	2.137 ± 0.09^g^	1.021 ± 0.02^b^	1.206 ± 0.02^b^	2.691 ± 0.03^ab^	0.677 ± 0.03^c^
NaCl (0 mM) + CQDs 5 mg L^− 1^	2.033 ± 0.08^g^	2.213 ± 0.08^ef^	1.035 ± 0.01^ab^	0.918 ± 0.06^de^	2.0918 ± 0.01^ef^	0.672 ± 0.03^c^
NaCl (0 mM) + CQDs 10 mg L^− 1^	2.123 ± 0.07^f^	1.766 ± 0.07^f^	0.976 ± 0.08^c^	1.202 ± 0.02^b^	2.375 ± 0.01^cd^	0.666 ± 0.01^d^
NaCl (0 mM) + Put-CQD NPs 5 mg L^− 1^	2.432 ± 0.07^cd^	2.001 ± 0.08^ef^	1.034 ± 0.08^ab^	1.215 ± 0.02^b^	2.552 ± 0.4^b^	0.687 ± 0.03^b^
NaCl (0 mM) + Put-CQD NPs 10 mg L^− 1^	2.731 ± 0.01^a^	1.564 ± 0.04^h^	0.975 ± 0.04^c^	1.749 ± 0.03^a^	2.748 ± 0.03^a^	0.698 ± 0.04^ab^
NaCl (100 mM) + No Treatment	2.052 ± 0.07^g^	3.677 ± 0.04^a^	0.861 ± 0.04^d^	0.558 ± 0.05^i^	1.328 ± 0.02^hi^	0.667 ± 0.01^d^
NaCl (100 mM) + Put 5 mg L^− 1^	2.227 ± 0.02^ef^	3.375 ± 0.06^ab^	1.028 ± 0.02^b^	0.659 ± 0.01^h^	1.746 ± 0.01^g^	0.696 ± 0.03^ab^
NaCl (100 mM) + Put 10 mg L^−1^	2.263 ± 0.08^e^	2.809 ± 0.03^bc^	1.071 ± 0.09^ab^	0.805 ± 0.02^fg^	1.985 ± 0.05^f^	0.695 ± 0.01^ab^
NaCl (100 mM) + CQDs 5 mg L^−1^	2.131 ± 0.09^f^	3.185 ± 0.07^ab^	1.138 ± 0.07^a^	0.669 ± 0.04^h^	1.272 ± 0.03^i^	0.692 ± 0.04^ab^
NaCl (100 mM) + CQDs 10 mg L^−1^	2.205 ± 0.05^ef^	2.953 ± 0.09^b^	0.867 ± 0.03^d^	0.746 ± 0.01^g^	1.343 ± 0.01^h^	0.675 ± 0.03^c^
NaCl (100 mM) + Put-CQD NPs 5 mg L^−1^	2.403 ± 0.06^d^	2.805 ± 0.07^bc^	0.986 ± 0.01^c^	0.855 ± 0.03^f^	2.037 ± 0.04^ef^	0.709 ± 0.02^ab^
NaCl (100 mM) + Put-CQD NPs 10 mg L^−1^	2.506 ± 0.09^b^	2.738 ± 0.05^c^	0.813 ± 0.06^e^	0.905 ± 0.06^e^	2.201 ± 0.03^e^	0.721 ± 0.02^a^

### Cellular damage indicators

**Fig. 3:**
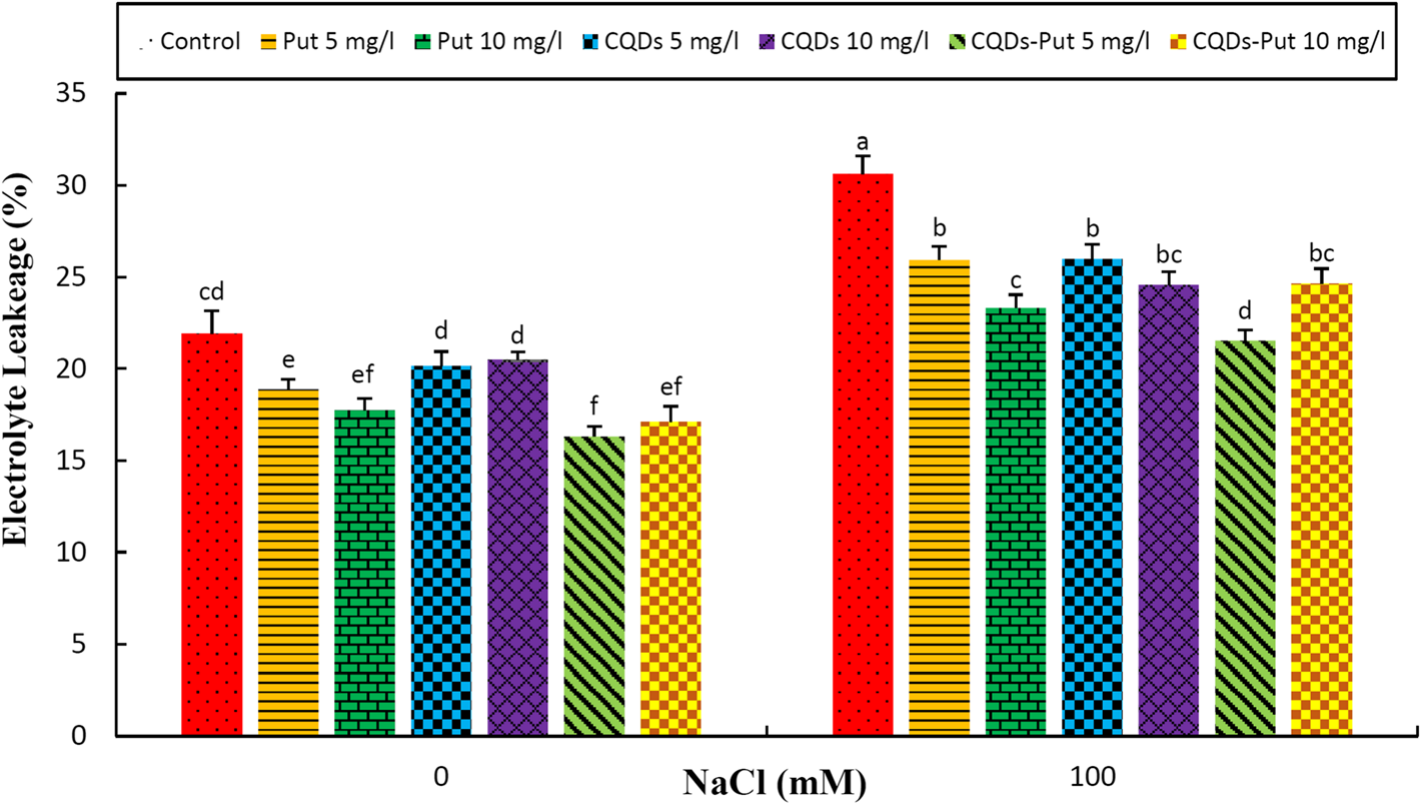
Effect of different concentrations of Put, CQDs, and Put-CQD NPs in electrolyte leakage (EL) of *Vitis vinifera* cv. ‘Sultana’ under salinity stress (CQDs, carbon quantum dots; Put, putrescine, Put-CQD NPs, carbon quantum dots functionalized by putrescine nanoparticles). Different letters indicate significant differences based on Duncan’s *post-hoc* analysis at *P* ≤ 0.05

Salinity caused significant enhancement (*P* ≤ 0.05) in electrolyte leakage (EL). All priming treatments significantly decreased EL values under both control and stress conditions with optimal protection being achieved following 5 mg L^− 1^ Put-CQD NPs priming treatment (Fig. 3).

**Fig. 4:**
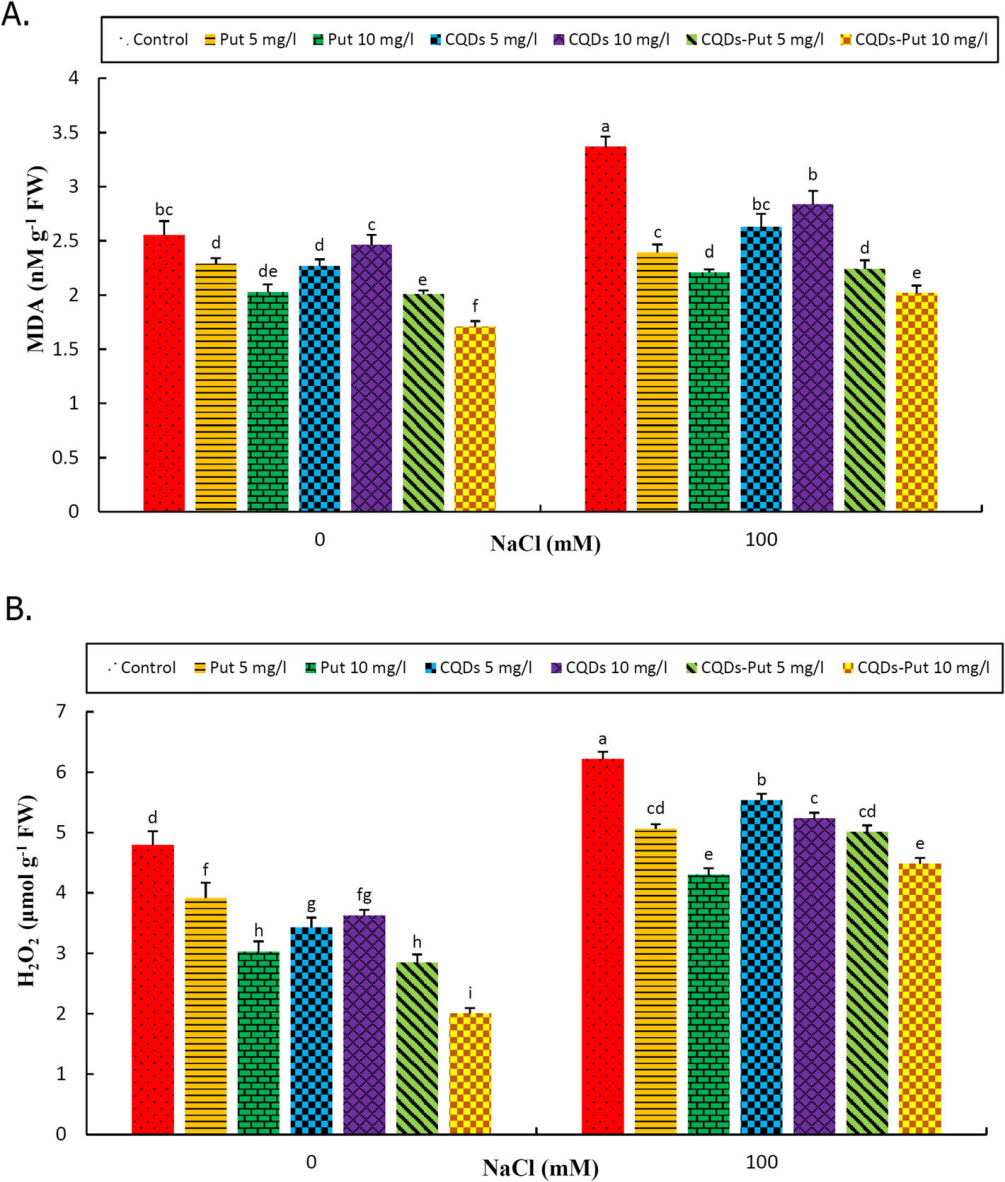
Effect of different concentrations of Put, CQDs, and Put-CQD NPs in MDA (**a**) and H_2_O_2_ (**b**) content of *Vitis vinifera* cv. ‘Sultana’ under salinity stress (CQDs, carbon quantum dots; Put, putrescine, Put-CQD NPs, carbon quantum dots functionalized by putrescine nanoparticles). Different letters indicate significant differences based on Duncan’s *post-hoc* analysis at P ≤ 0.05

As expected, MDA and H_2_O_2_ contents increased after imposing salinity stress, while priming treatments significantly decreased (*P* ≤ 0.05) MDA and H_2_O_2_ contents under both control and stress conditions. In general, 10 mg L^− 1^ Put-CQD NP priming treatment provided optimal results in terms of amelioration of MDA and H_2_O_2_ increases under both conditions (Fig. 4a, b).

### Proline and total phenolic compounds

**Fig. 5:**
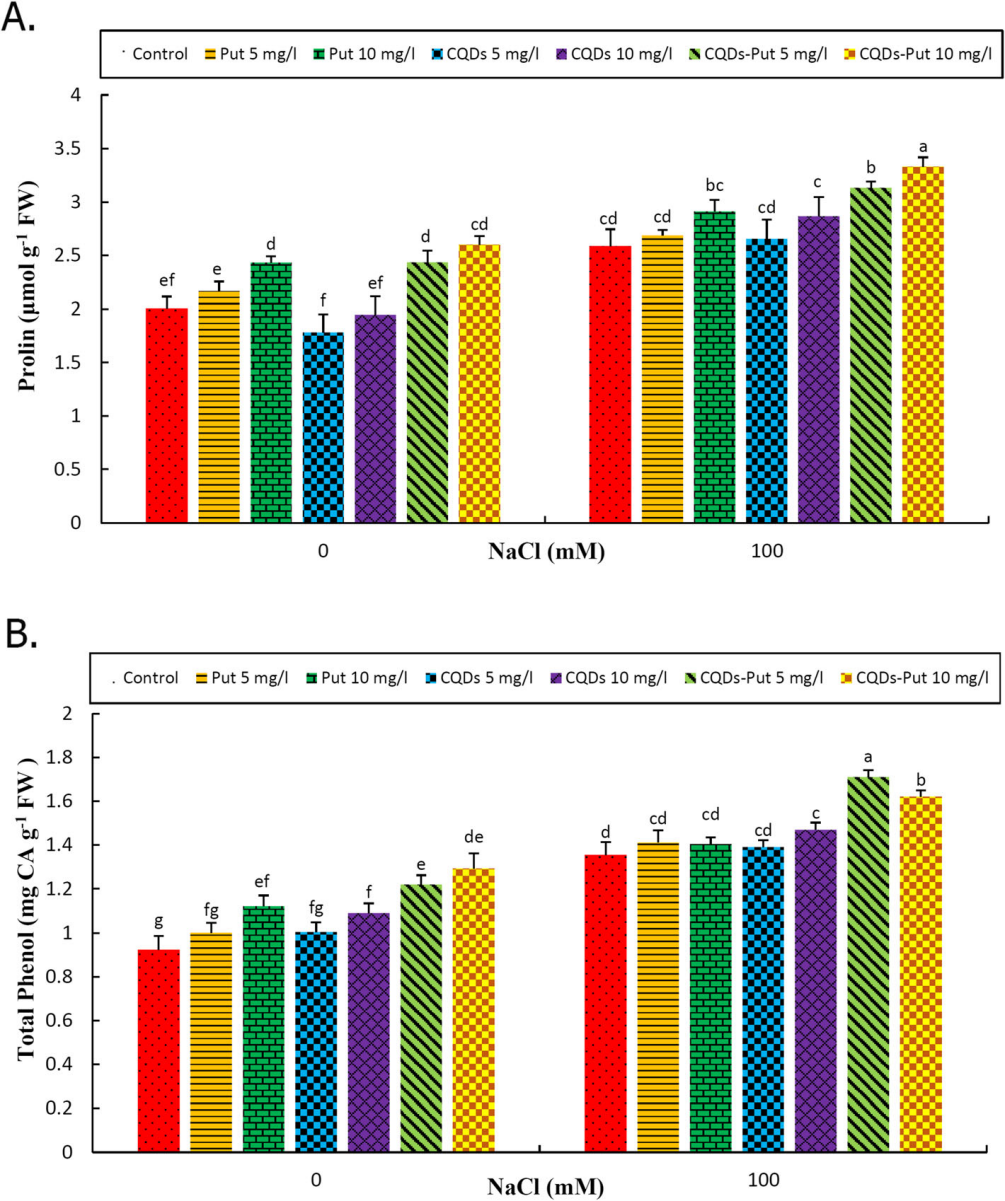
Effect of different concentrations of Put, CQDs, and Put-CQD NPs in proline (**a**) and total phenolic content (**b**) of *Vitis vinifera* cv. ‘Sultana’ under salinity stress (CQDs, carbon quantum dots; Put, putrescine, Put-CQD NPs, carbon quantum dots functionalized by putrescine nanoparticles). Different letters indicate significant differences based on Duncan’s *post-hoc* analysis at *P* ≤ 0.05

Salinity significantly enhanced (P ≤ 0.05) proline content and total phenolic compounds of grape (Fig. 5). Under control and stress conditions, priming treatments including Put at 10 mg L^− 1^ and Put-CQD NPs at 5 and 10 mg L^− 1^ concentrations significantly increased proline content, while the other treatment exerted no significant effect in this regard. The highest proline content was recorded following 10 mg L^− 1^ Put-CQD NP treatment under both conditions (Fig. 5a). Under control conditions, all priming treatments (with the exception of Put and CQDs at 5 mg L^− 1^ concentrations) increased total phenolics. Under salinity conditions, 10 mg L^− 1^ CQDs and 5 and 10 mg L^− 1^ Put-CQD NP priming treatments enhanced phenolic content; the other treatments had no effect compared with unprimed grapevine under salinity. The highest proline content was recorded following 10 mg L^− 1^ Put-CQD NP priming grapes under salt stress condition (Fig. 5b).

### Antioxidant enzymatic activities

**Fig. 6:**
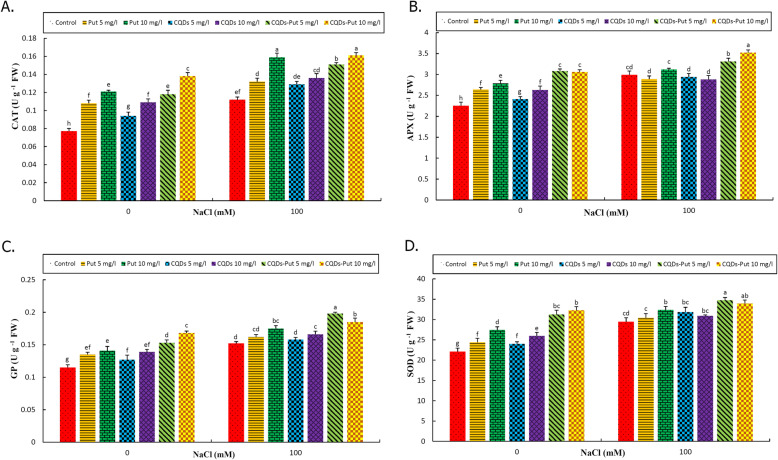
Effect of different concentrations of Put, CQDs, and Put-CQD NPs in antioxidant enzymatic activities of CAT (**a**), APX (**b**), GP (**c**), and SOD (**d**) of *Vitis vinifera* cv. ‘Sultana’ under salinity stress (CQDs, carbon quantum dots; Put, putrescine, Put-CQD NPs, carbon quantum dots functionalized by putrescine nanoparticles). Different letters indicate significant differences based on Duncan’s *post-hoc* analysis at *P* ≤ 0.05

Antioxidant enzymatic activities (CAT, APX, GP and SOD) were enhanced after imposing salinity (Fig. 6). All priming treatments increased CAT enzyme activity under control and stress conditions, with the highest activity of the enzyme being recorded following 10 mg L^− 1^ Put-CQD NP application, followed by Put at 10 mg L^− 1^ concentration under salinity conditions (Fig. 6a).

Considering APX enzyme, all priming treatments enhanced its activity under control conditions with optimal results following 5 and 10 mg L^− 1^ Put-CQD NP treatments. Under salinity conditions, only 5 and 10 mg L^− 1^ Put-CQD NP priming significantly increased APX activity, while other priming treatments had no significant effect compared with unprimed grapevines under salinity (Fig. 6b).

Under control conditions, all treatments increased GP enzyme activity with optimal activity being recorded following 10 mg L^− 1^ Put-CQD NP treatment. Most treatments, enhanced GP activity under salt stress, with the exception of 5 mg L^− 1^ Put and 5 mg L^− 1^ CQD treatments which demonstrated similar GP activity in unprimed grapevines under salinity. The highest activity was recorded following application of 5 mg L^− 1^ Put-CQD NPs (Fig. 6c).

SOD enzymatic activity increased significantly (*P* ≤ 0.05) following application of all priming treatments under control condition. The highest activity was recorded following 10 mg L^− 1^ Put-CQD NP treatment. Under salinity conditions, all treatments (except 5 mg L^− 1^ Put) enhanced SOD activity, with optimal results being recorded following 5 and 10 mg L^− 1^ Put-CQD NP priming treatments (Fig. 6d).

## Discussion

Salinity negatively affects plant growth and its physiological, metabolic and biological routes through induced osmotic stress [2]. Therefore, salinity decreases leaf FW and DW (e.g., [23]), in accordance with present findings. The positive effect of some NPs on agronomic traits under salinity stress has been previously reported (e.g., [12, 13]), in line with Put-CQD NPs effects. Priming with Put also exerted positive effects regarding leaf FW and DW correlating with enhanced salt tolerance of grapevine. Similar findings, supporting the protective role of Put or other PAs in plants under salinity stress, were previously reported ([23, 26, 28]). Put effects in improvement of growth parameters could be described via its polycationic nature and regulation of ion metabolism that enhance tolerance to salinity [28]. Therefore, positive effects of Put-CQD NPs could be attributed to the above-mentioned reasons.

In general, salinity enhances Na^+^ and reduces K^+^ ions in plants due to excessive amounts of Na^+^ in soil or nutrient solution that imbalance ion uptake by plants [27, 28, 31]. Beneficial effect of Put or other PAs on reducing Na^+^ and increasing K^+^ contents in plants under salinity was previously reported [23, 26]. This impact could be described through regulation of ion channel activity in root cells by PAs (e.g. Put) to repress Na^+^ influx into roots and enhance K^+^ influx from roots to shoots. In addition, reduction in the activity of plasma membrane-bound H^+^-ATPase via applied salinity could be alleviated by PA application, which then decreases Na^+^ and increases K^+^ contents in plants grown under salinity conditions [26]. Another possible reason for positive effect of Put in this regard might be through its role in stabilizing membranes and maintaining cation-anion balance [28]. Put acts as a signaling regulator responsible for inward rectification of K^+^ channels [28]. Mozafari et al. [31] reported positive effect of iron-NPs on decreasing Na^+^ and increasing K^+^ contents in plants under salinity conditions. To our knowledge, there is no prior art showing the effect of carbon-based NPs on Na^+^ and K^+^ contents in plants under salt stress conditions. This is therefore the first report demonstrating the positive effect of Put-CQD NPs on reducing Na^+^ and enhancing K^+^ contents in plants under salt stress conditions.

Salinity causes a drop in photosynthetic pigment content including chl *a*, *b* and carotenoids. Chl content declines through decrease in chl biosynthesis and increase in its degradation/turnover. Breakdown of photosynthetic pigments could occur via the accumulation of toxic ions in chloroplasts and enhancing oxidative stress in plants after imposing salinity stress [33]. In addition, inhibition of photochemical reactions and down-regulation of chloroplast-encoded genes due to salinity cause lead to a decrease in chl content [26]. Reduction in chl *a* and *b* content of plants under salinity stress conditions was noticed by Hatami et al. [33] and Gohari et al. [11–13], in accordance with the current study. PAs could reverse these negative effects of salinity by stabilizing oligomeric photosynthetic proteins and in particular the chl *a*/*b*-binding proteins displaying protease action during stress [26]. Although CQDs treatments enhanced chl *a*, *b* and total chlorophyll, further increase in CQDs concentration subsequently lowered their values [34]. Furthermore, Gohari et al. [12] reported positive effect of modified-MWCNTs in chl *a* and *b* content. The current study reported positive effect of CQDs in chl *a* and *b* content under salinity conditions. Salinity stress is known to alter biosynthesis and accumulation of secondary metabolites like carotenoids [35]. Decrease in carotenoids of grapevine under salt stress might be due to induction of the pathway for abscisic acid production in order to modulate plant growth [32]. Increased carotenoids improve plant tolerance to stress condition due to quenching of ROS and preserving chloroplast from photo oxidation under stress conditions through their non-enzymatic antioxidant function [36]. Positive effect of some NPs such as TiO_2_ and MWCNT NPs in carotenoid content has also been previously noticed in plants grown under stress conditions [12, 13], thus supporting the encouraging impacts of Put-CQD NPs in carotenoid content in grapevine under salinity conditions.

Chlorophyll fluorescence parameters are solid markers for the evaluation of physiological properties of plants and the detection of stress effects. A significant decrease in chlorophyll fluorescence parameters may be caused by the dissipation of a major proportion of light energy as heat under salt stress [37], previously reported by Netondo et al. [38] and Gohari et al. [12, 13]. Some studies indicated chlorophyll fluorescence adjustment following NP application [12, 13]. Increase in chl *a* and *b* could occur through enhancement in light energy of PSI absorbed by chloroplast membrane to be transferred to PSII, promotion of light energy conversion to electron energy and electron transport and acceleration of water photolysis and oxygen evolution [39]. Another probable reason might be the increased absorption of carbon dioxide in plants and RuBisCO enzyme activity with an important role in photosynthesis and chlorophyll fluorescence parameters [40].

Electrolyte leakage is a reliable cellular damage indicator that could identify any damage to cell membrane integrity [41]. Salinity increases EL value by disrupting cell membrane integrity. PA application lowered EL value of plants under salinity through their polycationic nature causing direct binding to negatively charged membrane phospholipid head groups that maintain membrane function and stabilized it under stress conditions [26, 28]. Furthermore, SWCNT and MWCNT application at lower doses decreased EL value and increased cell membrane stability under salinity condition [12, 33], while reduction in EL was also reported after SiO_2_ NP application [42].

ROS generation at high concentrations has destructive impacts such as lipid peroxidation that disturbs membrane integrity and enhances MDA content [43]. Increased MDA content was reported in plants under salinity. Exogenous application of PAs decreased MDA levels in plants under salinity [24, 26, 27, 44]. Furthermore, graphene QD application at low doses decreased MDA content [45]. Mozafari et al. [31] observed similar mitigating effect of iron-NPs on decreasing MDA content of grapevine under salinity. It is likely that stabilizing membrane integrity by Put and CQDs and their conjugated form (Put-CQD NPs) could (at least in part) justify the decrease in MDA content under salinity condition.

H_2_O_2_, a key regulator for multiple processes linked with growth, development and stress protection [46], has binary effects depending on its concentration. At low concentration, it acts as a signaling molecule needed for initiation of resistance mechanisms to biotic and abiotic stresses, while it results in oxidative stress [47] and programmed cell death when at high concentrations. H_2_O_2_ leads to lipid peroxidation through hydroxyl radical formation [48]. Increase in H_2_O_2_ content of grapevine under salinity was previously reported [31]. The observed decrease in H_2_O_2_ content following Put priming of plants under salinity comes in agreement with previous reports [24, 26]. This ameliorative effect could be described through the scavenging of free radicals and protection of proteins by PAs [28]. In fact, PAs could reverse salinity effects like ROS generation (e.g., H_2_O_2_), lipid peroxidation and corresponding MDA production [26]. Increase in proline by the treatments application could describe decrease in H_2_O_2_ values as increased proline could reduce H_2_O_2_ and other radicals either itself or by activating antioxidant enzymes activities (e.g., SOD, APX, GP and CAT) [49]. Mozafari et al. [31] reported that iron-NPs decrease H_2_O_2_ content in grapevine plants under salinity via increasing the antioxidant enzyme activities.

Proline is an osmolyte, metal chelating, antioxidant and signaling molecule [50]. Proline accumulates under abiotic stresses due to its role as osmotic regulator and ROS detoxifier that preserves membrane integrity, subcellular structures, antioxidant enzymatic activities and protein structure [31, 50]. Increase in proline content in salt-stressed grapevine has been previously recorded [31], likely due to a decrease in proline oxidation and increase in its biosynthesis [51]. Exogenous PA application including Put increased proline accumulation in plants under salinity stress [26, 44]. This enhancement could be considered as a mechanism to protect plants against salinity since proline is an osmolyte, storage material for nitrogen through stress, ROS scavenger and a modulator for NADP^+^/NADPH redox state. Increased proline content following PA application leads to protection of intercellular macromolecules, osmotic adoptability and the scavenging of hydroxyl radicals resulting in salt tolerance [26]. Iron-NPs were also shown to enhance proline content of grapevine under control and salinity conditions [31]. This enhancement was reported following QD application, as well [45], in accordance with the current study.

Phenolics protect plant cells through their potential to act as non-enzymatic and water-soluble antioxidants. This property is achieved via quenching of ROS and free radicals [52]. Phenolics prevent ROS generation and accumulation, thus inhibiting oxidative stress and reducing its undesirable effects [16]. Most phenolics are stimulated under biotic and abiotic stresses [49], such as under salinity conditions [52]. Feng et al. [45] reported increased phenolics following application of low concentration of QDs. In addition, MWCNTs-COOH treatment enhanced plant phenolics under salinity conditions [12]. Current results demonstrated positive effect of CQDs at 10 mg L^− 1^ and Put-CQDs at both concentrations probably via enhanced biosynthesis, as a line of antioxidant defense against oxidative stress imposed by NaCl. He et al. [53] demonstrated that PA application (spermidine) induced expression of genes related to enzymatic and non-enzymatic antioxidants (e.g. phenolics compound) in plants under salinity, in partial agreement with current findings.

SOD enzymatic activity eliminates superoxide radicals by dissimulating to H_2_O_2,_ which is then detoxified by several antioxidant enzymes (e.g. CAT, POD, APX, GP) [46]. CAT, as the main enzyme for H_2_O_2_ quenching, removeσ superoxide radicals as the first step of defense against ROS to reduce oxidative stress damage. APX removes H_2_O_2,_ similar to CAT, through the glutathione-ascorbate cycle [54]. GP enzyme utilizes glutathione to detoxify H_2_O_2,_ reducing lipids and organic hydroperoxides [46]. As salinity causes oxidative stress via ROS generation and accumulation in plant cells, antioxidant enzymes (such as CAT, SOD, GP and APX) could act as a defense mechanism for ROS detoxification. Accordingly, ROS quenching via antioxidant enzymatic activities reduces stress impacts, as an essential strategy for enhanced tolerance to stress conditions [26]. Increase in major antioxidant enzymatic activities has been well recorded in plants after imposing salinity [26, 54, 55], in agreement with current findings. PAs enhance the activity of antioxidant enzymes and non-enzymatic antioxidants (e.g. anthocyanins, flavonoids [23, 26];). In addition, PAs increase plant tolerance to salinity stress by scavenging free radicals of cells and improving cell survivability [24], as well as by inducing the expression of genes encoding antioxidant enzymes. Interestingly, PAs also act as direct free radical scavengers, due to PAs binding to antioxidant enzyme molecules [28]. Put prevents membrane peroxidation and denaturing of biomolecules under salinity through two mechanisms: First, extensive protonation of PAs at physiological pH enables them to scavenge free radicals directly or by conjugating to cell membrane; second, PAs by increasing antioxidant enzymatic activities and thus enhancing ROS detoxification and reducing oxidative damage. In total, these mechanisms lead to plant protection against salinity stress [23]. Such results were reported following exogenous application of PAs including Put on increased antioxidant enzymatic activities (SOD and CAT), leading to decreased ROS effects and membrane injuries [28]. In terms of the effect of nanomaterials, iron-NPs enhanced APX, SOD and POD enzymatic activities of grapevine under salinity conditions [31]. Feng et al. [45] reported enhanced CAT activity at lower graphene QD concentration, likely due to enhanced oxidative stress that reduces biosynthesis of antioxidant enzymes like CAT. Gohari et al. [12, 13] reported positive effect of MWCNTs-COOH and TiO_2_ NPs on SOD, CAT, APX and GP enzymatic activities under both control and salt stress conditions, in line with the current finding. Therefore, significant upregulation in all antioxidant enzymatic activities following Put-CQD NP treatment could ameliorate the negative impacts of salinity, demonstrating enhanced impacts of CQDs and Put in Put-CQD NPs.

## Conclusion

Taking into account the established effectiveness of Put as a stress-alleviating priming agent in plants and additionally introducing nanoparticle application as an innovative approach for improved delivery and efficiency of bioactive compounds, an advanced nanostructure was formulated using CQDs. Consequently, Put-CQD NPs were successfully applied as a priming treatment towards the improvement of grapevine cv. Sultana performance under salt stress conditions. Put-CQD NPs demonstrated improved effects compared with individual treatment of Put and CQDs, particularly at a concentration of 10 mg L^− 1^ through increase in a number of agronomic, physiological and biochemical parameters., highlighting a potential synergic effect of Put and CQDs in Put-CQD NPs. In conclusion, Put-CQD NPs represent an innovative approach that could be successfully applied in grapevines to improve performance under salinity conditions, while further validation is underway to determine their effectiveness in other crop species.

## Methods

### Experimental site, plant materials and applied treatments

The experiment was conducted in the research greenhouse of the Faculty of Agriculture, University of Maragheh, Maragheh, Iran (longitude 46°16′ E, latitude 37°23′ N, altitude 1485 m) as factorial experiment using a completely randomized design (CRD) in three replications. Two-year-old cuttings of grapevine cv. Sultana were planted in 7-kg pots containing a mixture of coco peat and medium grain perlite in a ratio of 3:1 (each pot contained a cutting). Then, they were irrigated with ½-strength Hoagland solution until at least eight true leaves emerged. At that point, plants were treated with the chemical priming treatments four times at 12 h intervals. The treatments included putrescine (Put) at two concentrations (5 and 10 mg L^− 1^), carbon quantum dot (CQD) NPs at two concentrations (5 and 10 mg L^− 1^) and putrescine-functionalized carbon quantum dots (Put-CQD NPs) at two concentrations (5 and 10 mg L^− 1^), each treatment in three replications. Treatments were done in combination with Hoagland solution into the culture medium of pots. The last application of priming treatments was performed 48 h prior to imposition of salt stress. Consequently, salinity stress at two concentrations (0 and 100 mM NaCl) was imposed daily through watering with Hoagland solution and continued up to a month. All biochemical and enzymatic measurements were implemented 3 days after imposition of salt stress using fully expanded leaves. Sampled leaves were instantaneously kept into liquid nitrogen for 2 min and afterwards preserved at − 80 °C freezer until measurements were carried out. Other parameters including Na^+^/K^+^ content, photosynthetic parameters and pigments were investigated a month after salinity application. Pigments were examined via the same above-mentioned sampling protocol, while leaf fresh and dry weights and photosynthetic parameters were assayed using fresh leaves. Three technical replications were used for each measurement. Control plants were irrigated simply with ½-strength Hoagland solution.

### Preparation of putrescine functionalized carbon quantum dots (put-CQD NPs)

In a 25 mL Teflon-lined autoclave chamber containing 10 mL distilled water, 0.5 g putrescine and 2 g citric acid were added and heated at 200 °C for 12 h. After cooling the reaction temperature to room temperature, the pH value of resulted red-brown solution was set to 7 by NaOH before use and characterization. For comparison, the same procedure was used to synthesize bare CQDs.

### Leaf fresh and dry weights

Five leaf samples were individually weighed for fresh weight (FW) and then kept in the oven (70 °C, 72 h) for dry weight (DW) measurements at the harvest stage.

### Na^+^ and K^+^ assay

Leaf samples were randomly collected from each treatment, washed and air dried and then dried in hot-air oven at 60 °C for 18 h. Afterwards, the samples were ground in Willey mill and the powered samples were stored for the assay. The triacid digestion extract was used for estimation of Na^+^ and K^+^ by flame photometry as out-lined by Ghosh [56] and expressed in mmol kg^− 1^. Thereby, Na^+^/K^+^ ratio was determined.

### Quantification of photosynthetic pigments (chlorophyll *a*, *b* and carotenoids)

Fully expanded leaves (0.2 g) were extracted in 0.5 mL acetone (3% v/v) and then centrifuged (10,000 rpm, 10 min) and the absorption of the obtained supernatant was recorded at 645 nm (Chl *b*), 663 nm (Chl *a*) and 470 nm (carotenoids) by UV-Vis spectrophotometry (UV-1800 Shimadzu, Japan). Chl *a*, *b* and carotenoids contents were calculated through the equations described by Sharma et al. [57].

### Chlorophyll fluorescence and SPAD assay

A dual-pam-100 chlorophyll fluorometer (Heinz Walz, Effeltrich, Germany) was used to measure chlorophyll fluorescence parameters including ^Fv^/_Fo_, ^Fv^/_Fm_ and Y (II). The measurement was done after the plants were dark-adapted for 20 min [58].

Five randomly selected leaves of each pot were used to determine SPAD values (leaf chlorophyll concentrations) via a SPAD-meter (502 Plus Chlorophyll Meter, Japan) [59].

### Electrolyte leakage (EL) assay

For EL assay, 0.5-cm diameter discs of fully expanded leaves were cut; the discs were then washed thrice by deionized water and incubated in ambient temperate for 24 h. A conductivity meter (Hanna, HI98192) was used to measure the initial electrical conductivity (EC1) of the solution. At that time, the samples were incubated in a water bath (95 °C, 20 min) to release all electrolytes, cooled down to 25 °C and their final electrical conductivity (EC2) was measured. The electrolyte leakage (EL) was calculated from following equation [60].

$$ \mathrm{EL}\ \left(\%\right)=\left(\mathrm{EC}1/\mathrm{EC}2\right)\times 100 $$

### Malondialdehyde (MDA) and hydrogen peroxide (H_2_O_2_) assay

After homogenizing 0.1 g leaf samples with 2.5 mL acetic acid (10% w/v) and centrifuging (15,000 rpm, 20 min), the same volume of the obtained supernatant and thiobarbituric acid (0.5% w/v) in trichloroacetic acid (TCA) (20%) was incubated at 96 °C for 30 min in the test tube. Samples were then placed at 0 °C for 5 min and centrifuged (10,000 rpm, 5 min) and the absorbance was recorded at 532 and 600 nm by the spectrophotometer. MDA content was calculated using the following equation:

$$ \mathrm{MDA}\ \left(\mathrm{nmol}\ {\mathrm{g}}^{-1}\mathrm{FW}\right)=\left[\left(\mathrm{A}532-\mathrm{A}600\right)\times \mathrm{V}\times 1000/\upvarepsilon \right]\times \mathrm{W} $$

Note: ɛ = the specific extinction coefficient (155 mM^− 1^ cm^− 1^), V = the volume of crushing medium, W = the leaf FW, A 600 = absorbance at 600 nm and A 532 = the absorbance at 532 nm [61].

To assay H_2_O_2_, 0.2 g leaves were finely mixed with 5 mL trichloroacetic acid (0.1% w/v) in an ice bath and then centrifuged (12,000 rpm, 4 °C, 15 min). To the obtained supernatant (0.5 ml), 0.5 mL potassium phosphate buffer (pH 6.8, 10 mM) and 1 mL potassium iodide (1 M) were added and the absorbance was recorded at 390 nm. Finally, H_2_O_2_ content was calculated by standard calibration curve previously made by various H_2_O_2_ concentrations and expressed as μmol g^− 1^ FW [62].

### Proline quantification

To assay proline content, 0.5 g leaf samples were homogenized in 10 mL aqueous sulfosalicylic acid (3%) in an ice bath. After centrifuging (1000 rpm, 4 °C), 2 mL ninhydrin acid and 2 mL glacial acetic acid (a 1:1:1 solution) were added to 2 mL supernatant, finely mixed and incubated at 100 °C for 1 h. The reaction was stopped in an ice bath and finally 4 mL toluene was added and mixed vigorously (20 s). The mixture absorbance was recorded at 520 nm using the spectrophotometer. Different concentration of L-proline was used for standard curve and final calculation of proline values [63].

### Quantification of total phenolic compounds

Briefly, after digesting 0.1 g leaf sample with 5 mL 95% ethanol, the mixture was kept in dark (24 h) and then to 1 mL of supernatant, 1 mL 95% ethanol and 3 mL distilled water were added. Next step was adding 0.5 mL 50% Folin-Ciocalteu solution and 1 mL 5% sodium bicarbonate, and after 1 h in the dark, the absorbance was recorded at 725 nm using the spectrophotometer. The absorbance values were converted to total phenols through standard curve made by different concentrations of gallic acid and expressed as mg gallic acid (GAE) g^− 1^ FW [64].

### Assay of antioxidant enzymatic activities

Total soluble proteins and antioxidant enzymes activities were assayed through leaves formerly stored at − 80 °C freezer. All steps of enzyme extraction were carried out at 4 °C as follows: leaves (0.5 g) were homogenized with potassium phosphate buffer (pH 6.8, 100 mM) containing 1% polyvinylpyrrolidone (PVP) and EDTA (4 mM) using magnetic stirrer for 10 min. After centrifuging (6000 rpm, 20 min), the supernatant was collected to evaluate total soluble proteins, catalase (CAT), ascorbate peroxidase (APX), superoxide dismutase (SOD) and guaiacol peroxidase (GP) enzymatic activities based on the same procedures described by Gohari et al. (2020b).

### Statistical analysis

All obtained data analysis was performed by SAS software and the means of each treatment were analyzed by Duncan’s multiple range test at the 95% level of probability (SAS Institute Inc., ver. 9.1, Cary, NC, USA).

## Data Availability

The data that support the findings of this study are available from the corresponding author upon reasonable request.

## Abbreviations

CQDs: Carbon quantum dots
Put: Putrescine
Put-CQD NPs: Carbon quantum dot functionalized by putrecine nanoparticles
EL: Electrolyte leakage
H_2_O_2_: Hydrogen peroxide
SOD: Superoxide dismutase
GP: Guaiacol peroxidase
APX: Ascorbate peroxidase
Chl: Chlorophyll
MDA: Malondialdehyde
CAT: Catalase
FW: Fresh weight
DW: Dry weight
MWCNTs: Multi wall carbon nanotubes
SWCNTs: Single wall carbon nanotubes
